# Course of objectively measured physical activity and sleep in postmenopausal breast cancer survivors during the COVID-19 pandemic: A 1-year follow-up

**DOI:** 10.3233/BD-230049

**Published:** 2023-12-20

**Authors:** Sandra J.M. van Cappellen-van Maldegem, Meeke Hoedjes, Michiel R. de Boer, Inge L. Nijman, Henrike M.W. van Valenberg, Jacob C. Seidell, Lonneke V. van de Poll-Franse, Laurien M. Buffart, Floortje Mols, Belle H. de Rooij

**Affiliations:** a Department of Medical and Clinical Psychology, Center of Research on Psychological Disorders and Somatic Diseases, Tilburg University, Tilburg, The Netherlands; b Department of Health Sciences and the Amsterdam Public Health Research Institute, VU University Amsterdam, Amsterdam, The Netherlands; c Department of General Practice and Elderly Care Medicine, UMCG, Groningen, The Netherlands; d Department of Developmental Psychology, Tilburg University, Tilburg, The Netherlands; e Department of Research & Development, Netherlands Comprehensive Cancer Organisation (IKNL), Eindhoven, The Netherlands; f Division of Psychosocial Research and Epidemiology, The Netherlands Cancer Institute, Amsterdam, The Netherlands; g Department of Physiology, Radboud Institute for Health Sciences, Radboudumc, Nijmegen, The Netherlands

**Keywords:** Physical activity, sleep, breast cancer survivors, COVID-19, accelerometer

## Abstract

**BACKGROUND:**

As physical inactivity and poor sleep quality may impose additional risk for cancer recurrence and overall mortality in postmenopausal breast cancer (PMBC) survivors, it is important to gain insight into the effect of the COVID-19 pandemic on their physical activity (PA) and sleep level.

**OBJECTIVE:**

This study aimed to assess the course of their physical activity (PA) and sleep throughout governmental measures against COVID-19 during 12 months of the COVID-19 pandemic.

**METHODS:**

PMBC survivors (*n* = 96) wore an ActiGraph wGT3X-BT for seven consecutive days at 12 and 18 months after diagnosis and additional measurements were taken after onset of the second (partial) COVID-19 lockdown. Longitudinal data was categorized into four timepoints: before onset of COVID-19 (T1), during the initial lockdown (T2), in between initial and second lockdown (T3), and during the second lockdown (T4). General linear mixed effects models assessed differences in moderate-to-vigorous physical activity (MVPA) per day, total minutes of PA per day, average acceleration, intensity gradient, sleep efficiency, and sleep duration over time.

**RESULTS:**

Levels of MVPA per day before COVID-19 were low (*Median* = 20.9 min/day (IQR = 10.8;36.2)), and time spent physically active was most often in light intensity, which remained stable throughout the pandemic. Sleep duration (*Median* = 442.8 min/night (IQR = 418.3;478.0)) and efficiency (85.9% (*IQR* = 79.6;88.4)) was sufficient before COVID-19 and showed stability over time.

**CONCLUSIONS:**

Low levels of PA with mostly light intensity, and adequate sleep efficiency and duration were observed before COVID in PMBC survivors. This was not further affected by COVID-19 governmental measures.

## 1. Introduction

The majority of postmenopausal breast cancer (PMBC) survivors have suboptimal lifestyle and body weight [1–3], poorer health-related quality of life, and an increased risk of type II diabetes mellitus, cardiovascular disease, second primary cancers, cancer recurrences, and all-cause mortality as compared with a healthy population [4–6]. These adverse health outcomes may partly be prevented by maintenance of healthy levels of physical activity (PA; i.e., at least 150 minutes spent on moderate intensity PA per week or at least 75 minutes spent on vigorous PA per week, or equivalent [7,8]). In addition, higher levels of PA are associated with lower risk of cancer recurrence and higher cancer survival [7,9]; reduced symptoms of anxiety and depression, and improved self-confidence [8,10].

In addition to being sufficiently physically active, it is important for PMBC survivors to maintain a healthy sleep duration (i.e., sleeping 7–9 hours a night [11]) and sleep quality. Both chronic sleep deprivation and excess sleep have been associated with early mortality in the general population [12]. Sleep deprivation may result in metabolic changes (e.g., altered endocrine function and glucoregulation, and increased inflammation) that may predispose women to breast cancer [13]. The influence of sleep on breast cancer prognosis is inconclusive, however it has been shown that sleeping nine or more hours per night is associated with an increased risk of breast cancer recurrence, and increased risks for both breast cancer-specific mortality and all-cause mortality [14]. In addition, poor sleep quality has been linked to negative emotions and mood, and an unhealthy lifestyle [15,16].

**Fig. 1.**
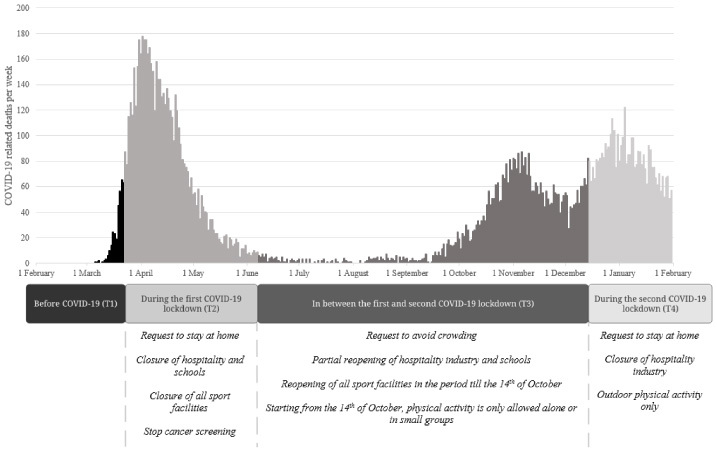
Timeline of reported COVID-19 related deaths per day, Dutch government COVID-19 measures, and study acquisition periods. Note. Timeline starting from 1 February 2019 till 1 February 2020. The number of inhabitants of the Netherlands is 17.51 million (3th of August 2021).

The Coronavirus disease 2019 (COVID-19) pandemic has a substantial impact on the lives of breast cancer survivors worldwide [17]. Until a sufficient degree of vaccination has been reached, hygienic means and physical distancing, including ‘intelligent’ lockdowns, are the most used strategies to minimize the progression and severity of the COVID-19 pandemic. In addition, the World Health Organization (WHO) strongly recommends social isolation for groups at risk of severe illness such as older or individuals with obesity and those with underlying health conditions [18]. In the Netherlands, the first partial lockdown took place between 23 March 2020 and 8 June 2020 and the second partial lockdown started at 15 December 2020 (see Fig. 1 for governmental measures during these partial lockdowns).

The COVID-19 pandemic, including COVID-19 lockdowns, may have impacted PA levels in PMBC survivors. Restrictions on ‘non-essential’ travel, the closure of gyms and sport courts, and the recommendation to stay at home may have decreased overall physical activity levels [19]. Moreover, the recommended social isolation for groups at risk of severe illness can make it extra challenging to meet physical activity guidelines [20].

Cancer survivors have shown to be worried about getting infected with COVID-19 more often as compared to a normative population [17]. Anxiety and uncertainties about health and economic security, may have negatively affected sleep onset and quality [21]. However, the lower number of social meetings may have resulted in longer sleep duration on weekdays and a decreased social jetlag (indicating less variable sleep timing comparing week and weekend days), positively affecting sleep quality [22]. In addition, during the second COVID-19 lockdown the COVID-19 pandemic already lasted for over 10 months, therefore possibly affecting psychological and emotional resources of cancer survivors. In contrast, in other PMBC survivors, the long period of COVID-19 pandemic may have led to adaptation and acceptance of the situation. Consequently, the effect of the initial and second COVID-19 lockdown on PA and sleep in these cancer survivors may differ.

As physical inactivity and poor sleep quality may impose an additional risk for cancer recurrence and overall mortality, it is important to gain insight into the effect of the COVID-19 pandemic on PA and sleep in PMBC survivors. To our knowledge, previous studies investigating the effect of COVID-19 lockdown on PA and sleep mainly focused on the general population, self-reported measures, and only during initial lockdown. This study aims to assess the course of accelerometer-measured PA and sleep during 12 months of the COVID-19 pandemic, and to gain insight into possible changes in PA and sleep of both the initial and second COVID-19 lockdown, in postmenopausal breast cancer (PMBC) survivors.

## 2. Methods

### 2.1. Participants

For the present analyses, data from the OPtimal TIming and Method for promoting sUstained adherence to lifestyle and body weight recommendations in postMenopausal breast cancer survivors (OPTIMUM)-study was used [23]. Details of the OPTIMUM-study are presented elsewhere [23].

**Fig. 2.**
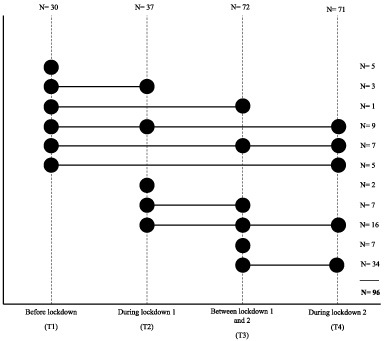
Overview of repeated measurements of physical activity and sleep using the Actigraph. Note. On the vertical axes, the number of observations per time point are depicted. On the horizontal axes, the number of participants per repeated measures timeline are depicted.

Patient recruitment and data collection for the OPTIMUM-study started in September 2019. Inclusion criteria were being diagnosed with breast cancer up to 6 months ago; being postmenopausal, having not menstruated for at least 1 year, and being able to read and understand Dutch. Each eligible PMBC survivor, from one of eight participating hospitals, was invited to participate in the study. The OPTIMUM-study is a longitudinal study with accelerometer measurements at 12 and 18 months following diagnosis. For the present analyses, we included data of the first 96 participants of the OPTIMUM-study who all had a first accelerometer measurement (at 12 months following diagnosis) between September 2019 and September 2020. Patients included in the OPTIMUM study between September 2020 and September 2022 are not included in the current analysis. Due to the continuous inclusion and measurement of the OPTIMUM-study, the time-point of the accelerometer measurements during the COVID-19 pandemic varied for all participating PMBC survivors The measurements were categorized into four time-periods: before onset of COVID-19 (T1), during the first COVID-19 lockdown (T2), the time in between the first and second COVID-19 lockdown (T3), and during the second COVID-19 lockdown (T4) (Fig. 2). Of the 96 participants, 30 had their first accelerometer measurement before the onset of COVID-19 (T1), 25 had their first accelerometer measurement during COVID-19 lockdown 1, and 41 had their first accelerometer measurement between lockdown 1 and lockdown 2 (Fig. 2).

Six months following their first accelerometer measurement, the PMBC survivors who were 18 months past their diagnosis, were invited for their second accelerometer measurement (*n* = 43). In addition, after onset of the second COVID-19 lockdown on the 15th of December 2021, we re-invited all 96 participants for an additional measurement with the accelerometer, of whom 71 (75%) participated. Due to the continuous inclusion and measurement in the OPTIMUM-study, the last measurement during the second COVID-19 lockdown occurred at varying timepoints since diagnosis (*mean* = 1.7 years since diagnosis (*SD* = 0.3 year)).

All participants were invited to wear the accelerometer at three time-points during this COVID-19 timeline. However, due to the continuous measurement and inclusion in the OPTIMUM-study not all participants had finished all measurements at the time of analyses. Additionally, there was missing data due to physical limitations (e.g., intensive breast cancer treatment; illness; broken leg) as well as practical issues (e.g., unpractical to wear the accelerometer in work setting, cleaning, healthcare including helping someone shower). In the present analyses we were able to include complete data for three out of four time-point during the COVID-19 pandemic for 32 PMBC survivors. For 42 PMBC survivors we could include data of 2 time-points, and for 14 PMBC survivors data of 1 time-point (Fig. 2). To assess the stability of our results, a sensitivity analysis was conducted of pairwise descriptive data of full responders across consecutive timepoints: T1 vs. T2 (N = 12), T2 vs. T3 (N = 23), and T3 vs. T4 (N = 58).

All participants provided written informed consent prior to the start of the study, and prior to the additional measurement during the second COVID-19 lockdown. The study protocol was approved by a medical ethical review board, according to the Dutch Medical Research Involving Human Subjects Act (WMO).

### 2.2. Procedure

A baseline questionnaire was completed 4–6 months after diagnosis (T0). Participants were invited to complete either an online or paper questionnaire via the PROFILES (Patient Reported Outcomes Following Initial treatment and Long term Evaluation of Survivorship) registry [24].

At 12 months and 18 months after diagnosis (during T1 or T2 or T3; varying per patient) and during the second COVID-19 lockdown (T4), participants completed follow-up questionnaires and wore an ActiGraph wGT3X-BT accelerometer (ActiGraph; ActiGraph, LLC, Pensacola, FL, USA) on their non-dominant wrist (i.e., defined as the hand they normally do not write with) [25]. All participants received a phone call to inform them about the procedure with respect to wearing the accelerometer, and a start-date was planned. We ensured that the participants had no planned vacation or other abnormalities during the planned week of wearing the accelerometer. Following, all participants received the accelerometer together with a paper instruction concerning device placement (illustrated in pictures), a wearing log, a sleep diary, and wear instructions via mail. Participants were instructed to wear the device 24 hours a day for 7 consecutive days, except during water-based activities such as showering, bathing, and swimming. Prior to distribution, each accelerometer was initialized to capture data at 100 Hz and was synchronized to Greenwich Mean Time.

### 2.3. Measures

#### 2.3.1. Sociodemographic and clinical characteristics

The baseline questionnaire assessed comorbidities in the past 12 months by use of the Self-administered Comorbidity Questionnaire (SCQ) [26]. The SCQ is a list of 14 medical conditions, with the option to list up to three additional medical conditions [26]. Follow-up questionnaires assessed body weight and height. Clinical data (i.e., cancer stage, date of diagnosis, and start- and end-date of treatment) was obtained from the Netherlands Cancer Registry (NCR). The NCR routinely collects data on all newly diagnosed cancer patients in the Netherlands [27]. Time since diagnosis was calculated by subtracting the date of diagnosis from the start day of wearing the accelerometer. Treatment during time of measurement was determined by subtracting the time since diagnosis from the number of days since treatment ended.

#### 2.3.2. Data processing and accelerometer-measurement of physical activity and sleep

Upon return of the accelerometer, the data was downloaded using the accompanying software ActiLife (Version 6.13.3; ActiGraph, Pensacola, FL, USA) and saved in raw format as .gt3x files. Subsequently, the .gt3x files were converted to time-stamp free .csv files which could be exported into R v.3.6.0. The .csv files were processed using the R-package ‘GGIR: Raw Accelerometer Data Analysis’ v.2.1-0 [28]. Data processing included detection of sustained abnormally high values, detection of nonwear time defined as the time not wearing the accelerometer in minutes [29], and auto-calibration of the raw tri-axial accelerometer signals using local gravity as reference [30]. In addition, within GGIR the Euclidean Norm Minus One (ENMO) (1g) was calculated averaged over 5 seconds epochs and expressed in milli-gravitational units (m*g*) as previously described by Rowlands and colleagues (2018) [31].

Data of participants was excluded from subsequent analysis if their accelerometer files demonstrated a post-calibration error larger than 0.01 g (10 m*g*); or if there were less than 3 valid wear-days (defined as ≥16 h per day) [31]. Detection of nonwear time has previously been described in detail (see “Procedure for nonwear detection” as supplementary document to van Hees et al. [29]). The default nonwear setting was used imputing invalid data by the average of similar time points on different days of the week [29], which means that for each valid wear day outcome variables were based on the complete 24 h cycle (1440 minutes) for all participants. PA level was expressed as average acceleration across the day (ENMO, m*g*) [31], average time accumulated in light intensity PA (LPA) per day (min/day), average time accumulated in moderate-to-vigorous PA (MVPA) per day (min/day), and time spent inactive per day (min/day). Time spent in LPA and MVPA was the accumulated time above an acceleration of respectively 50 m*g* and 100 m*g* [30]. In contrast, time spent inactive was defined as the time accumulated below an acceleration of 50 m*g* [31]. To determine the distribution of physical activity intensity by use of the intensity gradient (IG), the argument “iglevels = TRUE” was used in GGIR. The IG reflects the negative curvilinear relation between PA intensity and time spent in that intensity [31]. Periods of physical inactivity, defined as no changes in arm angle greater than 5 degrees for 5 minutes or more during a predefined nocturnal sleep window (e.g., from the participants’ sleep log), were classified as sleep [29,32]. Sleep quality was expressed as the sleep efficiency (i.e., proportion of the sleep span actually sleeping (%)), the frequency of long sleep interruptions (i.e., the number of long wake episodes ≥ 5 minutes), and average total number of minutes spent sleeping [32,33].

### 2.4. Statistical analysis

Descriptive information for the total group and for the participants at each time-point of normally distributed variables is presented as mean and standard deviation, otherwise the median and interquartile range are used.

Generalized linear mixed-effects models were built, using 3,000 parametric bootstraps, to investigate the changes in PA (MVPA per day; total min physical activity per day; average acceleration; intensity gradient) and sleep (sleep efficiency and sleep duration) over time. Time, the variable of main interest, was handled as categorical fixed effect including the four time points (i.e., before COVID-19 (T1); during the first lockdown (T2); in between the first and second lockdown (T3); during the second lockdown (T4)) in all models. All models included a random intercept to adjust for clustering of observations within participants. Time since diagnosis was entered as covariate in the basic mixed-effects models to adjust for the course of PA and sleep rehabilitation in the time since diagnosis till the first accelerometer measurement. In addition, adjusted mixed-effect models were built, extending previous models by adjusting for the following potential confounding background variables: age, receiving any active treatment versus no active treatment during the time of measurement (i.e., chemotherapy, radiotherapy, hormonal therapy or, targeted treatment; yes vs. no), number of comorbidities (0; 1; 2>), and employment. The models’ regression coefficients, 95% confidence intervals (CI), and *p*-values are presented.

All analyses were performed using R version 4.1.0 (https://cran.r-project.org).

## 3. Results

Participants (N = 96) were on average 64.3 (SD = 7.6) years old, 39% were employed, and mean BMI was 26.9 (SD = 4.3) (Table 1).

**Table 1:** Socio-demographic and clinical characteristics

	Overall	Before COVID^2^	During first COVID lockdown^3^	In between first and second COVID lockdown^4^	During second COVID lockdown^5^
		(T1)	(T2)	(T3)	(T4)
**N** ^1^	96	30	37	72	71
**Age, mean (SD)**	64.3 (7.6)	62.4 (7.5)	65.0 (8.2)	64.7 (7.2)	64.9 (7.9)
**Partner (yes), N (%)**	72 (67%)	20 (74%)	29 (85%)	55 (80%)	50 (75%)
**Education level, N (%)** ^6^					
Low	2 (2%)	2 (7%)	2 (6%)	0 (0%)	2 (3%)
Medium	55 (59%)	16 (59%)	24 (71%)	43 (62%)	39 (58%)
High	36 (39%)	12 (44%)	10 (29%)	26 (38%)	27 (40%)
**Employment (yes), N (%)**	36 (39%)	11 (41%)	13 (38%)	27 (39%)	24 (36%)
**BMI, mean (SD)**	26.9 (4.3)	26.8 (4.0)	26.6 (4.5)	27.1 (4.4)	Unknown^9^
**Comorbidities, N (%)**					
0	27 (28%)	11 (37%)	12 (32%)	19 (26%)	22 (31%)
1	28 (29%)	7 (23%)	10 (27%)	23 (32%)	18 (26%)
≥2	41 (43%)	12 (40%)	15 (41%)	30 (42%)	31 (44%)
**Tumor stage, N (%)**					
0	4 (4%)	2 (7%)	3 (8%)	3 (4%)	4 (6%)
I	47 (49%)	15 (50%)	17 (46%)	36 (50%)	36 (51%)
II	40 (42%)	12 (40%)	14 (38%)	29 (40%)	27 (38%)
III	4 (4%)	1 (3%)	3 (8%)	3 (4%)	3 (4%)
IV	1 (1%)	0 (0%)	0 (0%)	1 (1%)	1 (1%)
**Time since diagnosis (days), mean (SD)**	504.3 (128.7)	392.7 (57.7)	430.3 (80.5)	467.6 (91.6)	626.0 (108.4)
**Treatment during time of measurement** ^7^					
No treatment, N (%)	N/A^8^	8 (27%)	16 (43%)	27 (38%)	26 (37%)
Surgery (yes), N (%)	N/A	0 (0%)	0 (0%)	0 (0%)	0
Chemotherapy (yes), N (%)	N/A	0 (0%)	2 (5%)	0 (0%)	0
Radiotherapy (yes), N (%)	N/A	0 (0%)	0 (0%)	0 (0%)	0
Hormonal therapy (yes), N (%)	N/A	21 (70%)	19 (51%)	38 (53%)	38 (53%)
Targeted therapy (yes), N (%)	N/A	1 (3%)	1 (3%)	10 (14%)	9 (13%)

*Note*: Descriptives of socio-demographic and clinical characteristics for the total group and for the participants at each time-point. Due to repeated measures (see Fig. 2), the number of participants at the different time points does not add up to 96 (i.e., Total N).^1^ *N* = number of participants assessed by accelerometer per time point. Variables may deviate from 100% due to rounding off. *SD* standard deviation. *BMI* Body Mass Index. ^2^Measurement before 23 March 2020. ^3^Measurement between 23 March till 9 June 2020. ^4^Measurement between 9 June till 15 December 2020. ^5^Measurements between 15 December 2020 and 1 February 2021. ^6^Education level: Low (no or primary school); medium (lower general secondary education or vocational training); high (pre-university education, high vocational training, university). ^7^Treatment during the time of measurement with the Actigraph. Due to combined treatment in certain participants, percentages do not add up to 100%. ^8^N/A = not applicable. ^9^BMI was not measured during the second COVID lockdown.

All PA measures (min MVPA/day; total min spent physically active/day; average acceleration (m*g*); Intensity gradient) showed mainly engagement in low intensity PA and stable levels over the four time points (see Tables 2 and 3). The largest difference in min MVPA was observed between the first lockdown (T2) and the second lockdown (T4) (estimated difference 5.91 minutes per day; see Table 3). However, this difference was not statistically significant. A sensitivity analysis, conducted through pairwise descriptive analysis of full responders across consecutive timepoints (see Appendix), demonstrated consistent and stable results.

**Table 2:** Overview of physical activity and sleep parameters at all study time-points

	Before COVID^2^	During COVID lockdown 1^3^	In between COVID lockdown 1 and 2^4^	During COVID lockdown 2^5^
	(T1)	(T2)	(T3)	(T4)
**N**	30	37	72	71
**Physical activity**				
MVPA per day (min/day), *median (IQR)*	20.9 (10.8, 36.2)	14.8 (3.8, 31.1)	18.6 (5.1, 32.9)	26.8 (11.3, 37.1)
MVPA per week (min/week), *median (IQR)*	146.0 (75.7, 253.4)	103.6 (25.8, 217.5)	130.1 (35.4, 230.2)	187.3 (79.2, 259.9)
Light intensity physical activity (min/day), *median (IQR)*	117.7 (93.9, 140.6)	110.3 (90.3, 143.1)	113.2 (88.0, 142.8)	105.9 (82.5, 135.2)
Inactive time (min/day), *mean (SD)*	724.0 (93.3)	725.8 (89.0)	744.8 (75.0)	748.6 (69.0)
Average acceleration (mg), *median (IQR)*	22.0 (17.2, 25.0)	19.8 (16.1, 26.1)	21.3 (16.3, 25.3)	21.3 (18.2, 24.3)
Intensity gradient, *mean (SD)*	−3.4 (.3)	−3.4 (.4)	−3.4 (.4)	−3.4 (.4)
Total physical activity (min/day), *mean (SD)*	181.3 (60.8)	183.6 (79.4)	183.2 (69.3)	172.0 (55.6)
**Sleep**				
Sleep duration (min/night), *median (IQR)*	442.8 (418.3, 478.0)	454.4.0 (425.8, 489.9)	444.1 (396.4, 463.6)	453.2 (414.2, 484.7)
Sleep efficiency (%), *median (IQR)*	85.9 (79.6, 88.4)	87.5 (83.5, 91.5)	85.8 (82.8, 88.8)	86.3 (83.8, 90.6)
Frequency of sleep interruptions (*n*/night), *median (IQR)*	1.9 (1.5, 3.1)	1.8 (0.8, 3.5)	1.9 (1.4, 3.5)	1.9 (1.0, 3.4)

^1^Measurement before 23 March 2020. ^2^Measurement between 23 March till 9 June 2020. ^3^Measurement between 9 June till 15 December 2020. ^4^Measurements between 15 December 2020 and 1 February 2021. MVPA = moderate to vigorous physical activity; SD = standard deviation; IQR = interquartile range.

**Table 3:** Summary of mixed-effects analyses

	MVPA per day (min/day)					
	Basic model^*a*^			Corrected model^*b*^		
Time	*B*	*LL – UL 95% CI*	*p*	*B*	*LL - UL 95%CI*	*p*
*T2 vs. T1* (*N* = *67*)	−.49	−7.13; 6.55	.89	.93	−6.17; 7.53	.80
*T3 vs. T1* (*N* = *102*)	−.43	−8.76; 7.95	.92	1.07	−7.32; 8.76	.80
*T4 vs. T1* (*N* = *101*)	5.42	−6.23; 16.85	.36	7.76	−3.38; 18.76	.17
	Total physical activity (min/day)					
	Basic model^*a*^			Corrected model^*b*^		
Time	*B*	*LL – UL 95% CI*	*p*	*B*	*LL – UL 95% CI*	*p*

*T2 vs. T1* (*N* = *67*)	12.23	−8.14; 31.93	.25	10.15	−9.12; 29.63	.30
*T3 vs. T1* (*N* = *102*)	14.77	−11.64; 41.95	.27	15.10	−11.98; 41.03	.26
*T4 vs. T1* (*N* = *101*)	10.00	−26.06; 47.64	.57	11.07	−26.45; 46.85	.55
	Average acceleration (mg)					
	Basis model^*a*^			Corrected model^*b*^		
Time	*B*	*LL – UL 95% CI*	*p*	*B*	*LL – UL 95% CI*	*p*

*T2 vs. T1* (*N* = *67*)	−0.31	−2.64; 2.06	.80	−0.49	−2.91; 1.94	.68
*T3 vs. T1* (*N* = *102*)	−0.05	−2.42; 2.50	.96	−0.23	−2.89; 2.35	.86
*T4 vs. T1* (*N* = *101*)	0.06	−3.16; 3.37	.97	−0.10	−3.51; 3.39	.95
	Intensity gradient					
	Basic model^*a*^			Corrected model^*b*^		
Time	*B*	*LL – UL 95% CI*	*p*	*B*	*LL – UL 95% CI*	*p*

*T2 vs. T1* (*N* = *67*)	.04	−0.14; 0.22	.70	.01	−0.17; 0.19	.90
*T3 vs. T1* (*N* = *102*)	.04	−0.14; .21	.68	.03	−0.14; 0.20	.75
*T4 vs. T1* (*N* = *101*)	.10	−0.12; 0.32	.37	.07	−0.16; 0.29	.52
	Sleep duration (min/night)					
	Basic model^*a*^			Corrected model^*b*^		
Time	*B*	*LL – UL 95% CI*	*p*	*B*	*LL – UL 95% CI*	*p*

*T2 vs. T1* (*N* = *67*)	−5.70	−27.55; 15.80	.63	−2.91	−25.00; 20.48	.81
*T3 vs. T1* (*N* = *102*)	−16.01	−39.15; 6.36	.17	−11.76	−35.50; 12.76	.33
*T4 vs. T1* (*N* = *101*)	−13.19	−41.79; 15.58	.38	−6.27	−37.91; 23.88	.69
	Sleep efficiency (%)					
	Basic model^*a*^			Corrected model^*b*^		
Time	*B*	*LL – UL 95% CI*	*p*	*B*	*LL – UL 95% CI*	*p*

*T2 vs. T1* (*N* = *67*)	1.66	−0.84; 4.00	.19	1.46	−1.11; 4.08	.26
*T3 vs. T1* (*N* = *102*)	0.42	−2.16; 3.05	.75	.44	−2.14; 3.06	.75
*T4 vs. T1* (*N* = *101*)	1.41	−2.07; 4.94	.40	1.51	−1.83; 4.99	.39

*Note*: T1 = Before COVID; T2 = During first COVID lockdown; T3 = In between first and second COVID lockdown; T4 = During second COVID lockdown; MVPA = moderate to vigorous physical activity.^*a*^ Basic mixed-effect model for each of the main outcome measures, controlling for time since diagnosis (using 3000 bootstraps). ^*b*^Corrected model: adjusted for time since diagnosis, age, receiving active treatment at the time of measurement (chemotherapy, radiotherapy, hormonal therapy, targeted treatment), number of comorbidities (0; 1; 2>), employment.

Also, sleep duration and sleep efficiency was stable over the four time points (see Table 3). The largest difference in sleep duration was observed between the time before COVID-19 (T1) and the time following the first COVID-19 lockdown (T3) (estimated difference −16.01 min per day; 95%CI = −39.15;6.36; see Table 3).

Overall average relative nonwear time during waking hours was 1.2 % (SD = 1.9) and it was 12.7% (SD = 3.5) during the hours between sleep onset and wake onset. During the whole day average relative nonwear time was 5.5% (SD = 1.3). The average number of valid wear days was 5.8 (SD = .6), with an average of 1.1 weekend days (SD = .42) (data not shown).

Sensitivity analysis excluding the participants with only one Actigraph measurement in the COVID-19 timeline, provided comparable results.

## 4. Discussion

This study evaluated changes in PA and sleep during the COVID-19 pandemic in PMBC survivors. Overall, we observed that both PA and sleep seem stable over time. Although PA was relatively low at the first lock-down, no statistically significant differences were found over time. The stable PA levels and intensity we observed are in line with a previous study in the general population in the United Kingdom, showing that those aged >65 were able to remain physically active during and after the initial COVID-19 restrictions [34]. Similarly, in our sample with an average age of 64.3 years, the PMBC survivors maintained, on average, a stable level of minutes spent in MVPA per day after onset of COVID-19 restrictions.

However, as physical inactivity may impose an additional risk in the population of PMBC survivors for cancer recurrence and overall mortality, the observed low levels of PA spent in MVPA a day are alarming.

The low level of PA is comparable to other studies investigating cancer survivors prior to the pandemic [35]. Pooled data of eight studies in cancer survivors (using hip-worn Actigraphs) showed on average 26 min time spent in MVPA a day [35]. Our results indicated an average of 22.1 min before COVID-19 until the second COVID-19 lockdown, and 27.9 min a day during the second COVID-19 lockdown. Previous studies indicated that being older, having a higher BMI, and being female are associated with lower MVPA levels in cancer survivors [36]. In addition, compared to other studies, the measurements in our sample were relatively shortly following their diagnosis, and even included some individuals still receiving treatment during time of measurement (e.g., chemotherapy, hormonal therapy, and targeted therapy). This also may explain the low level of MVPA per day [37].

In addition, the intensity gradient of our sample indicates mainly engagement in low intensity PA. Most previous studies that demonstrated a health benefit of PA encouraged time spent in moderate or vigorous intensity PA [37,38]. It is uncertain whether engaging mainly in light PA could also be associated with lower levels of all-cause mortality and breast cancer recurrence, especially in case of high levels of sedentary behavior, which was shown to be common (66% of waking time) in breast cancer survivors [38,39].

As the majority of PMBC survivors have suboptimal lifestyle and bodyweight, they are at increased risk for second primary cancers and comorbid conditions compared to women without cancer [4–6,40]. The stability in the level and intensity of PA in our sample during the COVID-19 governmental measurements indicates that it did not worsen, yet their PA levels remained consistently low. Consequently, it is warranted to develop strategies to improve PA level in PMBC survivors. Hence, there is a need to investigate how to effectively and sustainably change PA behavior in this population.

The norm for sleep duration in Caucasian women aged 60 to 69 years old is 423.8 minutes a night [41]. In comparison, the average minutes spent sleeping in our study sample (mostly Caucasian women) was 445.6 per night indicating that overall, the participants did not experience major problems in sleep duration. Sleep duration and sleep quality were stable and sufficient in our sample of PMBC survivors throughout the COVID-19 pandemic. In relation to COVID-19, previous studies attributed poorer sleep quality partially to those experiencing higher levels of anxiety or depression during lockdown [42]. Even though it may be expected that cancer survivors are at higher risk for feelings of anxiety and depression during lockdown, it has been shown that cancer survivors reported almost similar levels of anxiety and depression as compared to a normative population [17]. This may contribute to the stable sleep parameters found in our study.

An important strength of this study is the use of accelerometer-measurement of PA and sleep. This may have circumvented reporting errors typically found while using self-reported measures (i.e., errors due to recall bias, misinterpretation, and social desirability) [43]. In addition, there was high compliance with daily wear time of the accelerometer. As a consequence, very little time needed to be imputed by the average of similar time points on different days of the week to add up to a complete 24-h cycle. Nevertheless, the study has several limitations. First, as the COVID-19 pandemic set in unexpectedly we could not control the number of observations at the different time points during the COVID-19 pandemic. This resulted in different trajectories of repeated measures across participants. The number of observations between the first and second COVID-19 lockdown was relatively high due to the long time span of this period in comparison to the other time points. In addition, the relatively large number of observations during the second COVID-19 lockdown can be explained by the invitation of all OPTIMUM-participants with a previous accelerometer measurement during the COVID-19 time span for this additional measurement. The different number of observations for the four time points and the different trajectories of repeated measures is taken into account by the use of mixed models but does result in relatively broad CI’s for periods with fewer participants. Our sample also precluded investigations into different trajectories of PA and sleep in subgroups of PMBC survivors. Second, some participants received active treatment (chemotherapy, hormonal therapy or targeted therapy) during any of the time points. Although we adjusted for active treatment in our analyses this may have affected the results. Specifically, PMBC survivors may have gradually increased their PA due to recovery from the diagnosis and subsequent treatment possibly balancing out a decrease in PA caused by COVID-19. Finally, only the baseline questionnaire included questions regarding the number of comorbidities.

The recommended level of PA holds spending at least 150 minutes on moderate PA per week or at least 75 minutes spent on vigorous PA per week. We found an average of 141.8 minutes spent on MVPA per week, indicating that a large proportion of the participants did not meet the PA recommendations. Additionally, a high proportion of PA was performed in lower intensity PA. As levels of PA were already low prior to the COVID-19 pandemic, it is not likely that the pandemic would have changed this behavior. Previous studies have shown that favorable lifestyle change following the cancer diagnosis is supported with evidence-based lifestyle counselling by oncology clinicians and health care practitioners concerning the benefits of sufficient PA for the health and prognosis of their cancer survivors [44]. Moreover, thus far oncology clinicians are found to be the most powerful catalysts for the promotion of health behavior [45,46]. Due to COVID-19 the number of follow-up consultations has diminished, and ongoing follow-up consultation was often replaced by telephonic or video consultation [17,47]. Consequently, possibly less counseling and advice to increase PA level may have occurred. Besides counselling, also referral to appropriate exercise-programs in line with clinical possibilities and preference may be challenging during the COVID-19 pandemic. Nevertheless, inactive patients could be advised to progressively replace sitting behavior with active breaks of walking at home and active patients to adopt physical activities to the home setting depending on ongoing COVID-19 measures.

In conclusion, on average, the PMBC survivors showed low levels of PA before COVID-19, and time spent physically active was mostly of light intensity. Sleep duration and sleep quality was sufficient before the COVID-19 pandemic in our sample of PMBC survivors. Our results point to stable levels of PA and sleep during the COVID-19 pandemic in PMBC survivors. As inactive and overweight PMBC survivors are at risk of worse progression of COVID-19, and at risk of cancer recurrence and worse prognosis following the diagnosis of cancer [20,47], it is important that health care professionals play a role in promotion of an active lifestyle for these patients, both during pandemics and thereafter.

## Data

The data that support the findings of this study are available on request from the corresponding author. The data are not publicly available due to privacy or ethical restrictions.

## Appendix

See Table A1.

**Table A1:** Sensitivity analysis: pairwise descriptive analysis of physical activity and sleep data of full responders for consecutive timepoints, T1 vs. T2 (N = 12); T2 vs. T3 (N = 23); and T3 vs. T4 (N = 58)

	Before COVID^2^	During COVID lockdown 1^3^	During COVID lockdown 1^3^	In between COVID lockdown 1 and 2^4^	In between COVID lockdown 1 and 2^4^	During COVID lockdown 2^5^
	(T1)	(T2)	(T2)	(T3)	(T3)	(T4)
*N*	N = 12		N = 23		N = 58	
**Physical activity**						
MVPA per day (min/day), *median (IQR)*	19.2 (13.5, 30.0)	6.0 (2.4, 25.2)	18.3 (8.9, 26.8)	16.4 (2.5, 33.3)	18.2 (9.7, 31.6)	29.0 (13.3, 37.5)
MVPA per week (min/week), *median (IQR)*	134.5 (94.2, 209.9)	42.0 (16.7, 176.1)	127.9 (62.0, 187.5)	114.9 (17.4, 233.3)	127.4 (68.0, 221.5)	203.3 (39.0, 262.8)
Light intensity physical activity (min/day), *median (IQR)*	117.9 (96.3, 140.6)	120.4 (90.8, 127.6)	99.3 (80.4, 155.5)	99.8 (84.4, 123.3)	118.1 (93.3, 144.7)	102.1 (81.2, 135.3)
Inactive time (min/day), *mean (SD)*	729.9 (84.9)	717.3 (84.9)	732.8 (94.9)	757.5 (97.0)	739.1 (72.2)	753.0 (65.0)
Average acceleration (mg), *median (IQR)*	22.2 (17.7, 24.8)	19.4 (16.2, 23.2)	19.5 (15.7, 26.3)	21.2 (16.2, 23.6)	22.7 (17.7, 25.4)	21.1 (18.2, 24.3)
Intensity gradient, *mean (SD)*	−3.4 (0.4)	−3.5 (0.5)	−3.4 (0.3)	−3.5 (0.4)	−3.4 (0.4)	−3.4 (0.4)
Total physical activity (min/day), *mean (SD)*	176.4 (56.6)	173.5 (70.9)	186.7 (86.9)	174.4 (76.7)	187.2 (70.1)	172.9 (55.8)
**Sleep**						
Sleep duration (min/night), *median (IQR)*	447.4 (422.1, 483.0)	447.3 (423.7, 455.5)	457.4 (428.2, 491.4)	454.3 (422.2, 487.1)	443.1 (396.4, 461.5)	444.8 (409.2, 473.7)
Sleep efficiency (%), *median (IQR)*	83.7 (81.0, 86.0)	85.9 (80.3, 88.0)	88.9 (83.9, 92.2)	86.7 (85.3, 87.8)	86.1 (82.8, 88.8)	86.2 (83.8, 90.0)
Frequency of sleep interruptions (n/night), *median (IQR)*	2.0 (1.5, 3.7)	1.9 (1.4, 3.9)	1.4 (0.4, 2.9)	1.9 (1.6, 2.5)	1.9 (1.4, 3.4)	1.9 (1.0, 3.4)

*Note:* T1 = Before COVID; T2 = During first COVID lockdown; T3 = In between first and second COVID lockdown; T4 = During second COVID lockdown; MVPA = moderate to vigorous physical activity. ^*a*^Basic mixed-effect model for each of the main outcome measures, controlling for time since diagnosis (using 3000 bootstraps). ^*b*^Corrected model: adjusted for time since diagnosis, age, receiving active treatment at the time of measurement (chemotherapy, radiotherapy, hormonal therapy, targeted treatment), number of comorbidities (0; 1; 2>), employment.
